# Mesenchymal stem/stromal cells in cancer therapy

**DOI:** 10.1186/s13045-021-01208-w

**Published:** 2021-11-17

**Authors:** Tianxia Lan, Min Luo, Xiawei Wei

**Affiliations:** Laboratory of Aging Research and Cancer Drug Target, State Key Laboratory of Biotherapy, National Clinical Research Center for Geriatrics, West China Hospital, Sichuan University, No. 17, Block 3, Southern Renmin Road, Chengdu, Sichuan 610041 People’s Republic of China

**Keywords:** MSC, Immunomodulation, Tumor-homing, Therapeutic mechanism, Signaling pathway, Anticancer strategy

## Abstract

The multipotent mesenchymal stem/stromal cells (MSCs), initially discovered from bone marrow in 1976, have been identified in nearly all tissues of human body now. The multipotency of MSCs allows them to give rise to osteocytes, chondrocytes, adipocytes, and other lineages. Moreover, armed with the immunomodulation capacity and tumor-homing property, MSCs are of special relevance for cell-based therapies in the treatment of cancer. However, hampered by lack of knowledge about the controversial roles that MSC plays in the crosstalk with tumors, limited progress has been made with regard to translational medicine. Therefore, in this review, we discuss the prospects of MSC-associated anticancer strategies in light of therapeutic mechanisms and signal transduction pathways. In addition, the clinical trials designed to appraise the efficacy and safety of MSC-based anticancer therapies will be assessed according to published data.

## Background

Mesenchymal stem/stromal cells (MSCs), which were first uncovered in 1976 [1], represent one of the most widely distributed cells in human body [2]. Generally, MSCs are characterized as multipotent stem cells that can differentiate into osteocytes, chondrocytes, adipocytes, and other lineages [3]. So far, MSCs have been isolated from various tissues including endometrial polyps, umbilical cord, menses blood, bone marrow, etc. Generally, human MSCs express markers including CD90, CD73 and CD105 [4]. However, the surface marker profiles of MSCs derived from different tissues are slightly different. For example, MSCs from bone marrow are positive for CD73, CD90, CD105, STRO-1 but negative for CD14, CD34, CD45 and HLA-DR. Except for those positive or negative markers described for bone marrow-derived MSCs, adipose tissue-derived MSCs are also positive for extra CD29, CD44, CD71, CD13, CD166, but negative for additional CD31. In addition, MSCs in peripheral blood express CD44, CD90, CD105, HLA-ABC but do not express CD45 and CD133. The detailed characters and immunophenotypes of MSCs derived from different tissues are summarized in Table 1.

**Table 1:** Biomarkers and characteristics of MSCs derived from different sources

MSCs isolated from different sources	Presence	Absence	Characteristics	References
Bone marrow	CD13, CD44, CD73, CD90, CD105, CD166, STRO-1	CD14, CD34, CD45, HLA-DR	Confirmed safety	[184–186]
			Capacity to differentiate into hepatocyte	
Adipose tissue	CD71, CD9, CD13, CD29, CD44, CD54, CD73, CD90, CD105, CD106, CD146, CD166, HLA I, STRO-1	CD14, CD19, CD31, CD34, CD45, CD133, HLA-DR	Abondance in adipose tissue	[184, 187–190]
			Have weaker differentiating potential towards osteocytes and chondrocytes	
Birth-derived tissues	CD29, CD44, CD73, CD90, CD105, CD146	CD14, CD34, CD45	Have relatively high proliferation rate	[191–193]
			Produce more insulin than bone marrow MSC	
Dental pulp	CD29, CD44, CD90, CD105	CD14, CD34, CD45	Have odontoosteogenic properties	[194–196]
			Locate within the dental crown	
Peripheral blood	CD44, CD54, CD90, CD105, CD166, HLA-ABC	CD14, CD34, CD45, CD31	Manifest similar immunophenotypes and differentiation potential to those of bone marrow MSC	[197–199]
			The volume of blood is large	
Endometrium	CD73, CD90, CD105, CD146	CD34, CD45	Have the potential of mesodermal lineage differentiation	[197, 200, 201]
			Producing high level of leukemia inhibitory factors	
Skin	CD44, CD73, CD90, CD105, CD166, CD29	CD34, CD45, HLA-DR	Have higher proliferation rate than that of adipose MSC	(202–204)

Notably, it has been well documented that MSCs have strong immunomodulatory effects and the capacities to migrate to inflammatory and tumor sites [5]. Equipped with immunomodulation capacity, MSCs play substantial roles in the regulation of immune responses and the development of a broad range of diseases [6]. As one of the most prevalent and fatal diseases, cancer is seriously threatening human health [7]. Importantly, it has been revealed that MSCs participate in the initiation, development, progression, and metastasis of cancer [8]. Moreover, since MSCs have tumor-homing property, they are considered as promising vehicles for the precise and selective delivery of anticancer drugs [9]. Recently, more mechanisms through which MSCs play promotive or suppressive roles on the development of cancers have been revealed [10], which led to the springing up of the explorations with respect to MSC-based anticancer therapies [11]. However, given the fact that MSCs are considered as a promising therapeutic tool for the treatment of cancer, some of its disadvantages, such as pro-metastasis functions [12] and the capacities to facilitate evasion of immune surveillance [12], are hampering the further clinical applications [10]. Hence, a comprehensive evaluation of MSC-based anticancer therapy on the basis of its therapeutic mechanisms and the underlying signal pathways is urgently needed for the purpose of accelerating the development of clinically applicable MSC-based anticancer cell therapies and improving quality of life for patients with cancer.

At present, the major sources of MSCs for clinical applications include bone marrow, cord blood, and adipose tissue. While cord blood could be obtained from birth-derived tissues, bone marrow and adipose tissue need to be procured using bone marrow aspirate or liposuction [13]. Moreover, density gradient centrifugation is commonly used to isolate MSCs from human tissues, and the isolated cells would be seeded on plastic culture plates to further exclude the hematopoietic cells, as hematopoietic cells do not attach to the plastic substrate [14]. The subsequent expansion and manufacturing of the MSCs should comply with the Good Manufacturing Practice (GMP) guidelines [15]. In this review, the potential anticancer therapeutic prospects of MSC-based approach will be evaluated in terms of the mechanisms and essential signaling pathways that are associated with the development of cancer. In addition, to facilitate the research and development of high-quality cell therapy drugs, the clinical trials designed to appraise the efficacy and safety of MSC-based anticancer therapies will be assessed according to published data.

## Controversial roles of MSCs in cancer

### Immunomodulation of MSCs

The ability of MSC to modulate immune response was first uncovered by Amelia Bartholomew et al. in 2002 [16]. They tested the ability to influence the proliferation of allogeneic lymphocytes of Baboon-derived MSCs in vitro and in vivo*.* Their data showed that Baboon MSCs could significantly suppress the proliferation rate of allogenic lymphocytes in vitro and prolong the survival of skin grafts in MHC-mismatched recipients in vivo. Inspired by this work, the immunomodulation effects of MSC have been extensively explored [17]. So far, apart from affecting lymphocytes proliferation, it has been shown that MSCs mediate immune response through a variety of ways, including inhibiting the activation of nature killer (NK) cells [18], repressing the activation as well as basic functions of dendritic cells (DC) [19], modulating proliferation and functions of B cells [20], and inducing the expansion of regulatory T cells [21]. Furthermore, the immunomodulatory potentials of MSCs are exerted by direct cell-to-cell contact or paracrine secretion of soluble factors [22]. It has been shown that the upregulation of cell adhesion molecules like galectin-1 and CD274 on MSCs not only enhanced the cell-to-cell contact, but also strengthened the immunomodulation effects [23, 24]. Besides, MSC could produce a wide spectrum of soluble factors such as cytokines, enzymes, and nitric oxide (NO) [25–27] to mediate the immune response.

Notably, the immunomodulation effects of MSCs associated with cancer therapies act in two opposite ways. On the one hand, MSCs are considered as a powerful component of the stem cell transplantation therapy. In particular, for the treatment of leukemia, multiple myeloma, and lymphoma, transplantation of allogenic bone marrow or hematopoietic stem cells (HSC) is one of the widely used therapies [28]. However, the allogenic transplantation could lead to graft-versus-host disease (GVHD), a major cause of morbidity and mortality in patients who received the treatment [29]. The immune suppressive functions of MSCs have been leveraged to relieve GVHD [30]. Furthermore, MSCs have been reported to facilitate the hematopoietic reconstitution following HSC transplantation [31]. Thus, MSC-based therapy represents a promising supportive method for HSC or bone marrow transplantation in patients with certain types of cancers. On the other hand, as a critical component of tumor microenvironment (TME) [32], MSCs contribute to the survival as well as proliferation of tumor cells [33] and inhibit natural anti-tumor immune response [34]. Hence, targeting MSCs in TME has been considered as a potential strategy to improve the outcomes of patients with cancer [35].

### Crosstalk between MSCs and tumor

Although the immunomodulation property is tightly associated with the growth of tumors, MSCs could directly impact cancer development through crosstalk with tumors mediated by cell-to-cell contact or secretion of soluble molecules [36]. It should be noted that, in terms of the treatment of cancer, the roles of MSCs are divergent [37]. Specifically, although numerous studies have demonstrated that MSCs have pro-tumor functions [38], it is also commonly accepted that MSCs could inhibit the growth of tumors through a multitude of mechanisms such as jeopardizing tumor cell cycle and inducing apoptosis [39].

On the one hand, MSCs could contribute to the development and progression of cancer through various ways. First, MSCs are able to influence the phenotype of tumor cells by secreted molecules. For instance, it has been reported that the C–C motif chemokine ligand 5 (CCL5) secreted by MSCs could increase the invasiveness of metastatic breast cancer cells [40]. Second, MSCs have been found to support tumor angiogenesis. For example, within TMEs of breast and prostate cancers, it was observed that the expression levels of angiogenic factors such as vascular endothelial growth factor (VEGF) and Interleukin 6 (IL-6) are upregulated by the MSCs. Consequently, these factors led to the enhanced tumor vascularization [41]. Third, accumulating evidence indicates that MSCs could be differentiated into cancer-associated fibroblasts (CAFs) in response to soluble factors produced by cancer cells. In particular, it has been demonstrated that transforming growth factor β1 (TGF-β1) derived from the TME of prostate cancer was capable of inducing the MSC-to-CAF differentiation [42]. Last but not least, apart from exerting pro-tumor properties with soluble signaling factors, MSC could more directly facilitate the growth of tumor through cell-to-cell contact or cell engulfment. Mele et al. have shown that, mediated by cell-to-cell contact, the surface-bound TGF-β of MSC promotes the epithelial-to-mesenchymal transition in colorectal cancer cells [43]. Besides, Chen et al. revealed that MSCs could be engulfed by breast cancer cells. More importantly, followed by the engulfment, the gene expression pattern of breast cancer cells was altered. Further, EMT, metastasis and invasiveness of breast cancer cells were found to be enhanced [44].

On the other hand, considerable number of studies converged on the finding that MSCs suppress the growth of tumor and progression of cancer. Previous investigations have unveiled the cytotoxicity of MSC on the tumor cells [45]. Additionally, a recent study has demonstrated that MSCs induce the apoptosis and suppress the proliferation of glioma cells through inhibiting phosphatidylinositol 3-kinase/protein kinase B (PI3K/AKT) pathway [46]. Notably, although it has been well documented that MSCs possess pro-angiogenic property, emerging evidence have shown that MSCs could also inhibit the tumor vascularization [47, 48]. Ho et al. have illustrated that MSCs repress the growth of tumor by inhibiting angiogenesis. Moreover, they further demonstrated that the inhibition of angiogenesis might be associated with the MSC-mediated downregulation of PDGF/PDGFR axis in glioma cells [49]. In addition, cell-to-cell contact is also implemented by MSCs to exert their anti-tumor effects. In a previous study, glioblastoma cancer stem cells (CSCs) were cocultured with umbilical cord derived MSCs (UC-MSCs). It was observed that the proliferation rate of cocultured CSCs was significantly reduced, which implied that such direct cell-to-cell interaction could lead to the inhibition of tumor growth [37]. Besides, Sarmadi et al. have shown that MSC arrests cell cycle in G0/G1 phase of lymphoid origin hematopoietic tumor cells by cell-to-cell contact [50].

In conclusion, the reported discrepancies with respect to the capacity of MSCs to inhibit or enhance cancer development might be consequent on the differences of experimental settings such as animal models, cell lines, doses, and duration times of treatment. Whether MSC should be considered as an anti-tumor agent or a therapeutic target for the treatment of cancer is still a matter of debate. Thus, more in vivo investigations should be conducted to more rigorously evaluate the tumor-inhibitory effect of MSCs. Furthermore, effective methods that could distinguish pro-tumor MSCs need to be developed to improve the specificity of targeted therapy.

### Signaling pathways regulated by MSCs

**Fig. 1:**
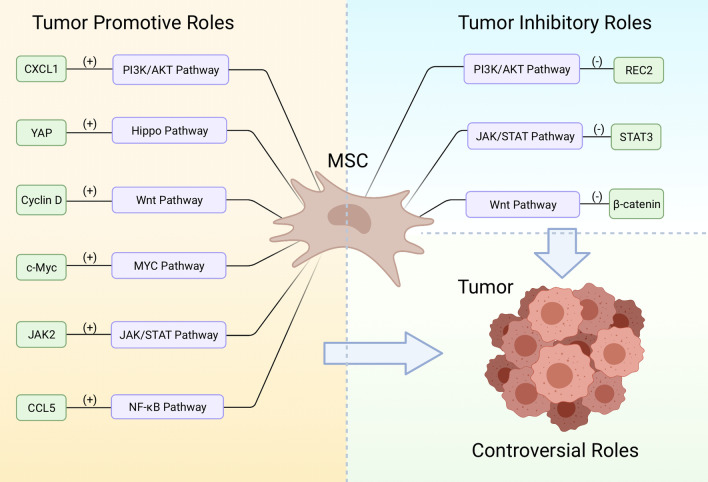
Schematic representation of the signaling pathways involved in the crosstalk between MSC and tumor. MSC play both pro-tumor and anti-tumor roles through upregulate or downregulate the activity of cancer-related signaling

The initiation, progression and development of cancer is associated with a wide spectrum of signaling pathways [51]. Compelling evidence suggests that the activities of some crucial cancer-related pathways are being up- or down-regulated by MSCs [52]. Hence, it is imperative to thoroughly scrutinize the signaling network that connects MSCs with cancer with the purpose of deeply accessing the potential anticancer effects of MSCs-based therapies. In this section, key cancer-related pathways affected by MSCs were selected for in-depth evaluation in regard to the pro- or anti-neoplastic effects (Fig. 1) according to published data.

#### PI3K/AKT signaling pathway

PI3K/AKT pathway is hyper- or hypo-activated in numerous types of cancers. These aberrations of PI3K/AKT pathway are coupled with the gaining of neoplastic properties by tumors, such as increased cell proliferation rate, drug resistance and stem-cell like phenotypes [53]. As a family of heterodimeric lipid kinases, PI3Ks are activated by various upstream factors, including cytokines, chemokines, antigens, and growth factors [54]. Notably, AKT, a serine/threonine protein kinase, is known as one of the most important effectors downstream of PI3K [55]. Moreover, PI3K/AKT pathway is linked with a huge number of signaling molecules and cascades that has been shown to participate in the development of cancers [56]. For example, it was shown that the inhibition of gene encoding PI3K catalytic subunit beta (PI3KCB) precipitated the reduction of cell proliferation rate and apoptosis in glioblastoma [57]. In thyroid cancer, the over-activation of PI3K/AKT pathway epigenetically suppresses the expression of the REC8 gene and inhibits the proliferation as well as colony formation capacity of cancer cells [58]. Additionally, Li et al. have revealed that the upregulation of PI3K/AKT pathway promotes the expressions of placental growth factor (PlGF) and C-X-C Motif Chemokine Ligand 1 (CXCL1) in lung cancer stem cells, which further stimulate angiogenesis [59]. CCPlenty of paracrine factors secreted by MSCs are ligands for receptors that activate PI3K/AKT pathway [52]. Thus, it is plausible that MSCs can influence the growth and metastasis of tumors via PI3K/AKT pathway. In a previous experimental study, it was suggested that the breast cancer-associated MSCs could facilitate the production of mammosphere and provide a “tumor-friendly” microenvironment via modulating the activity of PI3K/AKT pathway [60]. Besides, it was observed that the cell culture medium that had incubated bone marrow-derived MSCs for 48 h (MSC conditioned medium) significantly enhanced the progression of head and neck cancer through the activation of PI3K/AKT signaling pathway [61]. Therefore, based on the current knowledge, targeting PI3K/AKT might be an effective way to inhibit the pro-tumor effect of MSC.

#### JAK/STAT signaling pathway

The Janus kinase/signal transducers and activators of transcription (JAK/STAT) pathway are an evolutionarily conserved signaling cascade [62]. By regulating the activation of a quantity of functional molecules like growth factors and cytokines, JAK/STAT pathway closely links itself to a variety of developmental trajectories of tissues [63]. Consequently, the abnormal functioning of the pathway is associated with the development of various diseases, including cancer [64]. For instance, the upregulation of STAT3, a member of STAT family, is associated with the tumorigenicity of glioma stem-like cells [65]. In addition, it has been demonstrated that the deregulation of JAK3 promotes the invasiveness of extra-nodal nasal-type natural killer cell lymphoma [66]. Moreover, the elevated expression level of Stat5a/b has been considered as a predictive marker of recurrent prostate cancer [67].

Thus far, several studies have been conducted to evaluate how MSCs interact with tumors via JAK/STAT pathway. Nevertheless, with regard to the impacts on cancer development, divergent roles of MSC have been observed. Reportedly, IL-6 secreted by colorectal cancer-derived MSCs could activate JAK2/STAT3 signaling and promote the progression of colorectal cancer [68]. Additionally, in a recent study, it was observed that the chronic MSCs exposure contributed to the selection of metastatic prostate cancer cells that are more resistant to apoptotic effects, which is coupled with the alteration in IL-28/STAT signaling from the pro-apoptotic, IL-28-STAT1 cascade to the anti-apoptotic IL-28-STAT3[69]. Conversely, the anti-tumor effects of MSC mediated by JAK/STAT pathway have also been documented. He et al. have demonstrated that the MSC conditioned medium inhibits the STAT3 level in breast cancer cells and suppresses tumor progression, indicating that paracrine soluble factors secreted by MSC could modulate JAK/STAT signaling and inhibit the growth of breast tumor [70]. Hence, future studies should focus on the specific molecular mechanisms involved in the JAK/STAT-mediated interplays between MSC and tumors.

#### Wnt signaling pathway

Wnt pathway contains a large number of proteins that act as signaling molecules mediating tissue development and homeostasis [71, 72]. Besides, the pathway is also described as a key player regulating the development of cancer as well as the stemness of tumor cells [73, 74]. The roles that Wnt signaling plays has been well-studied in numerous types of cancers [75]. In colon cancer, it was elucidated that the secretion of Wnt ligands, such as Wnt3, by cancer cells contributes to the maintenance of Wnt activity [76]. In addition, Luis et al. have shown that the well-regulated Wnt signaling is crucial for sustaining the normal hematopoiesis, whereas its deregulation might precipitate the development of leukemia. In melanoma cells, it has been revealed that WNT5A, a Wnt protein, induces the release of exosomes containing pro-angiogenetic factors VEGF and IL-6[77]. Moreover, it is generally accepted that Wnt signaling is a key mediator of stemness in cancer stem cells [78]. Mechanistically, as a crucial signaling molecule in Wnt pathway, β-catenin improves the expression of telomerase reverse transcriptase (TERT), a ribonucleoprotein that prevents the loss of telomeres in cancer stem cells [79].

Although it was described that MSCs could modulate Wnt signaling in cancer [80], the question concerning whether such modulations inhibit or facilitate cancer development is still controversially discussed. In cholangiocarcinoma, Wang et al. revealed a pro-tumor effect mediated by human UC-MSCs [81]. They treated the xenograft tumor model established using cholangiocarcinoma cells with UC-MSCs and then observed that the expression levels of Wnt target genes such as cyclin D1 and c-Myc were upregulated. Moreover, the metastasis and chemoresistance of cholangiocarcinoma was also found to be enhanced following the treatment of UC-MSCs. In contrast, the report from another group suggests that MSC secretes dickkopf-related protein 1 (DKK-1), a soluble Wnt antagonist, to modulate Wnt signaling and decreases the proliferation rate of leukemia cancer cells [82]. Hence, MSC-mediated Wnt signaling changes might play dual roles in cancer progression, this discrepancy might be attributable to the differences in cancer types, sources of MSC, and the activation status of Wnt proteins.

#### Hippo signaling pathway

The Hippo pathway play central roles in cell proliferation and growth of organs [83]. In recent years, there has been accumulating evidence suggesting that the pathway contribute to the progression of cancer [84]. The network of Hippo pathway consists of multiple proteins, including Yes-associated protein/WW-domain-containing transcription regulator (YAP/TAZ), large tumor suppressor 1/2 (LATS1/2) and mammalian Ste20-like kinases 1/2 (MST1/2) [85]. Notably, as a tumor suppressor, the activation of Hippo pathway is linked with the inhibition of YAP/TAZ activity. Hence, YAP/TAZ has been considered as an oncogene as it was found to be overexpressed in a large number of cancers [86, 87]. Dysregulation of the Hippo pathway contributes to tumorigenesis. In cholangiocarcinoma, overexpression of YAP is associated with metastatic disease and poor prognosis [88]. In addition, Hippo pathway was found to be suppressed during the development of colorectal cancer, which further led to the tumor metastasis [89].

With respect to MSC-based cancer therapies, very limited evidence indicates that MSCs can regulate the Hippo pathway to affect the neoplastic process [90]. However, it has been demonstrated that MSC secretes prostaglandin E2 (PGE2) to activate YAP in liver cells and promote the proliferation of hepatocytes as a result [91]. Furthermore, Liu et al. have shown that, under hypoxia condition, MSCs contribute to the growth of hepatocellular carcinoma via a similar molecular mechanism [92]. However, more investigations need to be conducted for acquiring a more comprehensive understanding of the role of Hippo pathway in the crosstalk between MSC and tumors.

#### MYC signaling pathway

MYC is a family of genes that encodes three transcription factors: c-Myc, n-Myc, and l-Myc. Among them, c-Myc is characterized as an important oncogene and is considered as an ideal therapeutic target in anticancer therapy [93]. The stimulatory roles of MYC on cancer progression have been well studied [94, 95]. For example, it has been reported that overexpression of c-Myc induces the EMT in mammary cells, which could potentiate the motility of cancer cells. Moreover, high expression level of c-Myc in patients with diffuse large B-cell lymphoma is reported to be coupled with compromised overall survival [96]. More importantly, in more than 70% of human cancers, the abnormal expressions of c-Myc were observed [97].

In recent years, a few reports indicate that MSC regulate the progression and drug resistance of cancers through MYC signaling pathway. Particularly, it was revealed that Galectin 3 expressed by MSCs in the TME of acute myeloid leukemia (AML) activates MYC expression and contributes to the cell adhesion between MSC and AML tumor, thereby promoting the survival of cancer cells [98]. Besides, it was observed that the bone marrow MSC conditioned medium enhances the growth of gastric tumor via upregulation of c-Myc [99]. In addition, in a previous study, it was demonstrated that cell-to-cell contact between MSC and AML cell prevents cancer cells from apoptosis and fosters the drug resistance of AML [100]. Hence, it is plausible that c-Myc could be considered as a promising target for anticancer therapies, as the inhibition of the pathway not only directly suppress the development, but also interrupts pro-tumor pathways governed by MSC.

#### NF-κB signaling pathway

Nuclear factor kappa B (NF-κB), a transcription factor family, was first uncovered by Sen et al. in 1986 [101]. The family comprises five members: RelA, RelB, c-Rel, NF-κB1/p50 and NF-κB2/p52, all of which participate in the regulation of the expression of a variety of genes [102]. In the progression of cancer, the NF-κB signaling pathway plays considerable roles [103]. For example, in an early study, the suppression of NF-κB signaling has been found to reduce the incidence of colitis-associated cancer [104]. Moreover, in breast cancer, it was shown that the activation of NF-κB signaling contributes to EMT and tumor metastasis. Similarly, in gastric cancer, the upregulation of NF-κB signaling is associated with the improvement of tumor invasiveness and is also considered as a prognostic marker in patients with gastric cancer [105].

Compelling evidence suggests that a part of pro-tumor effect of MSC is mediated by NF-κB pathway. For instance, it was unveiled that, in acidosis condition, IL-6 secreted by cancer-associated MSCs upregulates NF-κB and fosters the stemness as well as chemoresistance of osteosarcoma cells [106]. Furthermore, Wu et al. have shown that the MSC conditioned medium could induce the activations of mTOR and NF-κB signaling in colorectal tumor and facilitate the cancer progression [107]. In addition, MSC in inflammatory environment upregulates NF-κB signaling following activation of the C–C chemokine ligand 5/C–C chemokine receptor type 5 (CCL5/CCR5) axis, which plays pro-metastatic roles [108]. Thus, it is conceivable that inhibition of NF-κB is an effective approach to repress the pro-tumor effect of MSC.

## The tumor homing property of MSCs

Since the development of cancer shares multiple similarities with wound healing, tumors are also known as “wounds that do not heal” [109]. Biological processes such as the growth of new blood vessels, activation of fibroblasts, remodeling of extracellular matrix (ECM) are associated with both tumor growth and wound healing [110]. Importantly, it has been demonstrated that MSCs are capable of homing to injured or inflammatory sites [111]. In 2003, MSCs were found to spontaneously distributed in a variety of tissues following systemic infusion in baboons, suggesting that MSCs could selectively migrate to certain locations [112]. Then, in 2004, Jeanmarie et al. demonstrated that MSCs could engraft in the tumor sites in the gastric cancer model [113], indicating the tumor homing capacity of MSCs.

However, although it has been observed that infused MSCs could be home to tumor sites in various types of cancers [114], the underlying mechanisms are still unclear. Based on current knowledge, the tumor homing capacity of MSCs is mainly mediated by the cooperation of cytokines, chemokines, and other functional molecules [115]. Firstly, cytokines are involved in the adhesion and traversing process of the vascular endothelial layer of circulating MSCs. It has been shown that tumor necrosis factor alpha (TNF-α), IL-6, IL-1β and interferon-gamma (IFN-γ) contribute to this process [116–118]. Secondly, chemokines could stimulate chemotaxis in cells, which leads to the migration of cells to targeted tissues or sites [119]. For instance, stromal cell-derived factor-1 (SDF1) is highly expressed on the surface of tumor cells [120]. It was observed that MSCs could express CXC chemokine receptor 4 (CXCR4), a receptor of SDF1. And the CXCR4-SDF1 axis plays important role in the induction of MSCs migration towards tumors [121]. Thirdly, other functional molecules such as growth factors and hypoxia-inducible factor (HIF) were also found to be the mediators of tumor homing of MSCs. While growth factors like platelet derived growth factor (PDGF) could affect the transmigration capacity of MSCs [122], HIF could induce the expression of CXCL10, and its cognate receptor, CXC chemokine receptor 3 (CXCR3), on MSCs and breast cancer cells, respectively [123]. Although the mechanisms whereby MSCs migrate to tumor sites are still not comprehensively understood, the tumor tropism of MSCs has been leveraged to develop more specific and effective anticancer therapies [124].

## Current strategies in MSC-based cancer therapy

The intricate pattern of interaction between MSCs and tumor makes researchers remain cautious about using MSCs in the anticancer therapies. However, the quick development of gene engineering techniques makes it possible to load the agents with the well-established anti-tumor effects into MSCs using viral vectors, non-viral vectors or other transfection tools [125]. Furthermore, the tumor tropism of MSCs allows them to precisely release the drug near the tumor site, which, theoretically, increases the safety and efficacy of the treatment. In addition, a growing number of studies have shown that MSC-derived exosomes can be utilized as powerful cell-free cancer treatment.

### MSC as a vehicle for therapy delivery

**Fig. 2:**
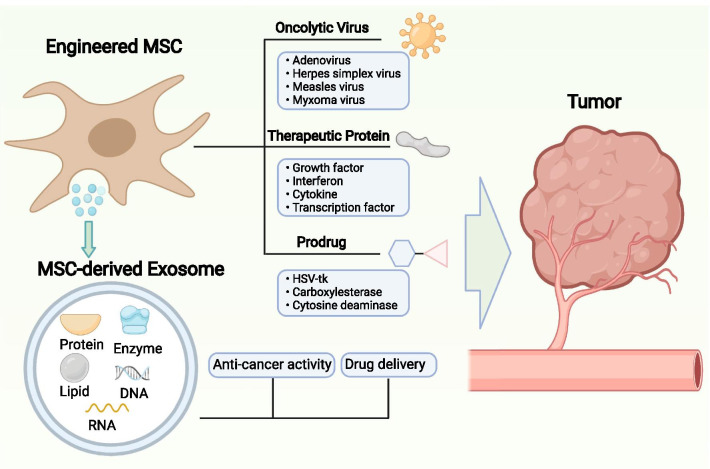
Schematic illustration of the current antitumor therapies based on MSC and MSC-derived exosome

As a vector of anti-tumor drugs, MSCs could be genetically modified to express or secret a variety of agents that suppress the growth and progression of cancer [126]. These agents could be classified into three major groups: therapeutic proteins, suicide genes, oncolytic viruses (Fig. 2).

#### Delivery of therapeutic proteins

A variety of proteins such as cytokines [127, 128] and growth factors [129] have been identified as potent regulators of the development of cancers. Therapeutic proteins that suppress the tumor growth or act as inhibitors of pro-tumor factors represent a novel form of anticancer drug. MSCs are considered as ideal vehicles for the delivery of such proteins. For example, interferons are considered as potent anti-tumor agents because they have been shown to inhibit the proliferation of tumor cells and modulate the immune response[130]. The strategies linked IFN-β with tumor-specific antibodies [131] or traditional chemotherapy drugs [132] have been shown to efficiently control the progression of cancer in animal models. Reportedly, MSCs that had been genetically edited to produce IFN-β showed anti-proliferative and proapoptotic effects on tumor cells [133]. Moreover, IL-12 has been considered as another promising protein for immunotherapy against cancer, as it stimulates the activation of T cells and cytotoxic NK cells [134]. It was shown that IL-12-expressing MSCs could inhibit the growth of tumors in both renal cell carcinoma and cervical tumor models established in mice [135, 136]. Kanehira et al. have demonstrated that MSCs that express NK4, an inhibitor of hepatocyte growth factor (HGF) could effectively suppress the progression of lung metastatic tumor [137]. Besides, the engineered MSCs that could secrete a variant of thrombospondin (TSP)-1, a protein that inhibit angiogenesis, remarkably suppressed the vascularization at tumor site and led to the reduction of tumor growth rate in a glioma model [138]. However, it was revealed that (TSP)-1 accelerates the proliferation rate of MSCs through TGF-β signaling [139]. Thus, the safety of this therapy needs to be rigorously evaluated since the stem cell transplantation is usually associated with the potential risks of teratoma formation [140]. Additionally, the anticancer roles of MSCs that express a pro-apoptotic protein, tumor necrosis factor-related apoptosis induced ligand (TRAIL), have been analyzed in a series of investigations. The data show that, in a wide range of cancers including lung, breast, brain, cervical and colorectal cancers, TRAIL-expressing MSCs induced the apoptosis in neoplastic cells [141–143].

#### Delivery of suicide genes

Except for expressing anticancer cytokines, MSCs have been engineered to secret suicide genes that could convert nontoxic reagents into toxic anti-tumor drugs [144]. For instance, MSCs expressing a “suicide gene,” cytosine deaminase::uracil phosphoribosyltransferase (CD::UPRT), are efficient for inhibiting tumor growth in a prostate cancer model [145]. CD::UPRT can convert the non-toxic 5-fluorocytosine into the toxic anti-tumor drug 5-fluorouracil [146]. MSCs secreting CD::UPRT have also been shown to be effective in models of colon cancer and melanoma established in mice [147, 148]. In a more recent study, Liu et al. were inspired by the correlation between tumor and tissue stiffness and developed a precise drug delivery system called mechanoresponsive cell system (MRCS) using MSC. To establish the system, MSCs were modified to express YAP/TAZ and a suicide gene encoding cytosine deaminase (CD) [149]. Reportedly, YAP/TAZ are important mediators that could sense the stiffness of tissues [150]. As tumor and metastasis sites are associated with extensive collagen linearization which leads to increased matrix stiffness [151], YAP/TAZ in MRCS that homed to tumor would be activated and then stimulate the expression of CD. This system has been evaluated in the model of lung metastasis of breast tumor and manifested good efficiency in inhibiting the metastasis with minimal side effects [149].

#### Delivery of oncolytic viruses

Besides, oncolytic viruses (OVs) which could selectively kill cancer cells are also considered as one of the prospective anticancer agents [152]. By targeting the surface proteins on cancer cells, OVs recognize and bind to cancer cells, leading to oncolysis [153]. In a recent study, it was observed that oncolytic herpes simplex virus-1 modulates the TME via reducing the percentage of anti-inflammatory macrophages and increasing the number of tumor-infiltrating lymphocytes. In addition, the combination of oncolytic herpes simplex virus-1 and immune checkpoint modulators was found to significantly prolong the survival of the tumor-bearing mice [154]. However, the efficiency of the direct administration of OV is usually low because the defense mechanism of host can eliminate the exogenous viruses [155]. Therefore, MSCs were implemented as vectors to transport and shield OVs [156]. Moreover, the tumor-homing property of MSCs could increase the specificity of drug delivery, thereby further avoiding the potential attacking of normal tissues. In animal models, it has been shown that the MSC-mediated administration of OVs was effective for treating malignant glioma, hepatocellular carcinoma, and lung metastases of breast carcinoma [157–159]. Except for cytokines, suicide genes and OVs, some other agents such as growth factor antagonists, pro-apoptotic proteins, and antiangiogenic agents were also considered as potential therapeutic proteins that could be over-expressed by MSC after genetical modifications [160–162].

To conclude, tumor-homing capacity represents one of the most important therapeutic mechanisms of MSCs that has been utilized to develop precision medicines for the treatment of cancer. This inherent property of MSCs makes it possible to specifically deliver multiple types of anticancer agents to pathological regions. However, there are still several potential issues associated with such therapy. First, after the eradication of neoplastic cells, the remaining engineered MSCs may cause unexpected problems [163]. Second, the therapeutic proteins carried by MSCs may affect the proliferation of the transplanted MSCs and increase the risk of teratoma formation. Third, the efficaciousness of the tumor homing of MSCs is sometimes limited by the insufficient expression of homing molecules, which might lead to off-target issues [5].

### MSC-derived exosomes

Exosomes are a type of nanoparticles generated by cellular multivesicular bodies, with a diameter size about 50-200 nm [164]. Moreover, exosomes are globally secreted by all types of cells and abundantly exist in the body fluids [165]. As important signal transducers between cells, exosomes contain a considerable level of biologically functional molecules such as DNAs, enzymes, proteins, RNAs, and lipids (Fig. 2), which play different roles in the interactions among cells via a variety of mechanisms[166]. Thus, it is conceivable that cell-derived exosomes could be leveraged for therapeutic applications. Notably, MSC is one of the most efficient human cell types in the production of exosomes [167], which makes MSC-derived exosomes be considered as a prospective approach to treat diseases, including cancer [168]. Pascucci et al. found that MSC-derived exosomes could uptake Paclitaxel (PTX) after priming MSCs with PTX, indicating that MSC-derived exosomes is a novel method for drug delivery [169]. Besides, it was reported that the delivery of therapeutic microRNA, anti-miR-9, to chemo-resistant glioblastoma multiforme by MSC-derived exosomes was more efficient than that through direct intercellular communications between MSCs and cancer cells [170]. Further, MSC-derived exosomes have also manifested anti-angiogenesis activities. In 2013, Lee et al. showed that exosomes derived from MSCs could inhibit angiogenesis through downrgulating the expression of VEGF in breast cancer model [171]. Recently, a group from Tehran University demonstrated that exosomes from dental pulp MSCs that over-express miR-34a, a tumor suppressor microRNA, could inhibit the growth of tumor in vitro. Recently, Zhou et al. have demonstrated that exosomes from bone marrow-derived MSCs could induce the anti-tumoral macrophage polarization and elicit the recruitment of cytotoxic lymphocytes, thus enhancing the immunotherapy against pancreatic cancer in vivo [172]. In contrast, MSC-derived exosomes was reported to contributing the development of chemoresistance of breast cancer. After treating mice with doxorubicin (a standard chemotherapy drug), the expression level of MSC-derived exosome has been observed to be increased and subsequently induced the expression of S100A6, an anti-apoptotic factor, in breast cancer cells [173].

Taken together, MSC-derived exosome therapy is a promising approach to further improve the efficacy and safety of traditional anticancer therapies. Emerging evidence suggests that exosomes from MSC are superior drug deliver method in terms of gene transfer capacity, biocompatibility, immunogenicity, and stability [174]. Nevertheless, the complicity of the contents within the exosomes makes it a strenuous job to completely detangle the mechanisms by which exosomes interact with tumors. Moreover, future investigations may focus on developing gene engineering techniques that could modify the surface and content of exosomes to increase the targeting specificity [175].

## MSC-based therapies in clinical trials

**Fig. 3:**
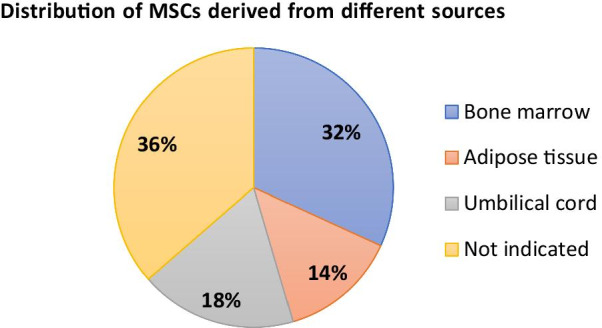
Illustration of the distribution of MSCs derived from different sources in clinical trials

Although it is clear that MSC is tightly linked with the cancer development [176], the lack of tools for specifically defining the heterogeneous MSC populations [177] and the contradictory relationship between MSC and tumor [178] have impeded the development of MSC-based therapies for oncological applications. So far, 31 clinical trials accessing the MSC-based therapies for the treatment or alleviation of cancer conditions have been registered on *ClinicalTrials. Gov* database. Among them, 16 trials are aimed to use MSCs for the treatment of cancer, 7 trials are using engineered MSCs as vehicles of therapeutic cytokines or oncolytic virus, 1 trial is designed to evaluate the safety and efficacy of MSC-derived exosomes with KrasG12D siRNA (iExosomes) in pancreatic cancer (Table 2) Furthermore, according to the database, 7 trials used bone marrow-derived MSCs, 3 trials used adipose tissue-derived MSCs, 4 trials used cord blood-derived MSCs, and 8 trials did not indicate the source of MSCs. (Fig. 3).

**Table 2:** Clinical studies using MSC-based therapies for cancer treatment

Form of drug	Cancer applications	Interventions	Phase	NCT NO
Tissue-derived MSC	Hematopoietic and lymphoid cell neoplasm	Cord blood MSCs	I/II	NCT04565665
	Acute leukemia	IFNγ-primed bone marrow MSCs	I	NCT04328714
	Pancreatic cancer	Adipose MSCs	I	NCT04087889
	Mandible tumor	Adipose MSCs	I/II	NCT03678467
	Acute respiratory distress syndrome (ARD) in patients with malignancies	Bone marrow MSCs	I	NCT02804945
	Myelodysplastic syndromes	Cord blood MSCs	I/II	NCT03184935
	Rectal cancer	NeuroRegen Scaffold™ + Cord blood MSCs	I/II	NCT02648386
	Hematologic malignancies	MSCs	I	NCT02181478
	Prostate cancer	Bone marrow MSCs	I	NCT01983709
	Expanding umbilical cord blood derived blood stem cells for treating leukemia, lymphoma, and myeloma	Bone marrow MSCs	I/II	NCT01624701
	Solid organ cancers	MSCs	I	NCT01275612
	Hematological malignancies	Cord blood transplantation + MSCs	I/II	NCT01092026
	Leukemia, lymphoma, and myeloma	Hematopoietic stem cells + MSCs	II	NCT01045382
	Bone neoplasms	Pre-immunodepleted MSCs	II/III	NCT00851162
	Myelodysplastic syndromes	Cord blood MSCs	II	NCT01129739
Engineered MSC	Ovarian cancer	MSCs secreting INF-β	I	NCT02530047
	Adenocarcinoma of lung	MSCs secreting TRAIL + standard therapy	I	NCT03298763
	Diffuse intrinsic pontine glioma	Radio therapy + Bone marrow MSCs infected with oncolytic virus	I	NCT04758533
	Glioma	Bone marrow MSCs infected with oncolytic virus	I	NCT03896568
	Head and neck cancer	MSCs secreting IL-12	I	NCT02079324
	Ovarian, primary peritoneal or fallopian tube cancer	Adipose MSCs infected with oncolytic virus	I/II	NCT02068794
	Metastatic and refractory tumors	Bone marrow MSCs infected with oncolytic virus	I/II	NCT01844661
MSC-derived exosome	Metastatic pancreas cancer	MSCs-derived exosomes with KRAS G12D siRNA	I	NCT03608631

Although a big proportion of trials were designed to evaluate MSCs that are not genetically modified, no data have been published from such trials registered on *ClinicalTrials. Gov* database. Theoretically, the administered MSCs could potentially modify the immune response or interact with tumors via cell–cell contact and secretion of soluble factors, thereby playing anticancer roles. However, considering the pro-tumor effects of MSCs in TME and the controversial roles that MSCs play in the interactions with cancer cells, the administration of unmodified MSCs might not be an efficient way to treat cancers.

Equipped with the tumor-trophic migration property, the genetically modified MSC could home to tumor site to deliver anti-tumor agents. In clinical studies, MSCs are used as vectors of cytokines and oncolytic virus. To date, the results from two of such trials have been published. First of which has shown that the bone marrow MSCs infected with ICOVIR5, an oncolytic adenovirus, was well tolerated in patients with relapsed/refractory pediatric solid tumors. In addition, two of nine patients who received the treatment showed disease stabilization [179]. As the first clinical trial using genetically modified MSCs in patients with cancer, the result of this study indicates favorable safety and quality of the therapy. The second trial with published data was conducted in Germany. It is a phase I/II study evaluating the safety and efficacy of MSCs that express thymidine kinase of the herpes simplex virus (HSV-Tk) in patients with advanced, recurrent, or metastatic gastrointestinal or hepatopancreatobiliary adenocarcinoma [180]. It was reported that the treatment was safe and tolerable. Furthermore, the median time to progression and median overall survival was 1.8 months and 15.6 months, which indicate the preliminary stabilization of the disease [181]. Both trials described above used MSCs as vectors of oncolytic virus. Comparing to therapeutic proteins like cytokines and growth factors, the anticancer effect of the oncolytic virus is clearer. Additionally, combining the MSC-based anticancer therapies with traditional chemo- or radio-therapies is also an ideal option that could improve the efficacy of current strategies. However, in another clinical trial with published data, the bone marrow-derived MSCs failed to manifest antitumor effects in patients with prostate cancer. Unfortunately, the results from the clinical study suggest that the MSCs did not home to the primary tumors [182]. Therefore, the feasibility of tumor-tropism of MSC might differ from cancer to cancer. Besides, the source of MSC and the route of administration may also affect the efficacy of the genetically modified MSCs.

The application of MSC-derived exosomes is one of the current hot topics in the research of cell-free therapies [183]. For the treatment of cancer, one active phase I clinical trial is designed to assess the therapeutic value of iExosomes in pancreatic cancer with KrasG12D mutation. In iExosome, the MSC-derived exosomes load the siRNA against KrasG12D, thereby specifically inhibiting the activity of KrasG12D in patients. MSC-derived exosome is a promising platform to further improve the anticancer effects of traditional therapies, and it is expectable that more translational studies will be conducted to further reveal its therapeutic potentials.

## Conclusions

MSC-based therapies are emerging as an attractive option for the treatment of cancers. As a matter of fact, MSC can modulate the immune response to neoplastic diseases and home to tumor sites. Furthermore, mediated by a wide array of signaling pathways, the interactions between MSCs and tumors are involved in the induction or inhibition of cancer progression and metastasis. However, the discrepancies regarding the impacts of MSC on cancer development remain largely unexplored, which has, to a large extent, hindered the transitions of bench-to-bed MSC-based applications. Up to date, a few clinical studies have been registered to analyze the therapeutic values of tissue-derived MSCs, engineered MSCs, and MSC-derived exosomes. Encouragingly, favorable clinical outcomes indicating safety and efficacy have been obtained from several trials. In conclusion, although the development of MSC-based anticancer therapies is still relatively slow and the functions of unmodified MSCs in the treatment of cancer are still not clear, promising progress have been made in clinical studies, especially for those designed for assessing the efficacy and safety of genetically altered MSCs. Future studies may focus on the strategies that take advantage of the anti-tumor properties while circumvent the pro-tumor effects of MSCs.

## Data Availability

Not applicable.

## Abbreviations

MSC: Mesenchymal stem/stromal cell
NK: Natural killer
DC: Dendritic cell
NO: Nitric oxide
HSC: Hematopoietic stem cell
GVHD: Graft-versus-host disease
TME: Tumor microenvironment
CCL: C–C motif chemokine ligand
CCR: C–C chemokine receptor type
VEGF: Vascular endothelial growth factor
IL: Interleukin
CAF: Cancer-associated fibroblast
TGF-β1: Transforming growth factor β1
PI3K/AKT: Phosphatidylinositol 3-kinase/protein kinase B
CSC: Cancer stem cell
PI3KCB: PI3K catalytic subunit beta
PlGF: Placental growth factor
CXCL: C-X-C Motif Chemokine Ligand
YAP/TAZ: Yes-associated protein/WW-domain-containing transcription regulator
LATS1/2: Large tumor suppressor 1/2
MST1/2: Mammalian Ste20-like kinases 1/2
PG: Prostaglandin
TERT: Telomerase reverse transcriptase
UC: Umbilical cord
DKK-1: Dickkopf-related protein 1
JAK/STAT: Janus kinase/signal transducers and activators of transcription
CC: Colorectal cancer
NF-κB: Nuclear factor kappa B
AML: Acute myeloid leukemia
ECM: Extracellular matrix
SDF1: Stromal cell-derived factor-1
TNF: Tumor necrosis factor
IFN: Interferon
HIF: Hypoxia-inducible factor
HGF: Hepatocyte growth factor
TSP: Thrombospondin
TRAIL: Tumor necrosis factor-related apoptosis-induced ligand
CD::UPRT: Cytosine deaminase::uracil phosphoribosyltransferase
MRCS: Mechanoresponsive cell system
OV: Oncolytic virus
PTX: Paclitaxel
iExosomes: MSC-derived exosome with KrasG12D siRNA
HSV-Tk: Thymidine kinase of the herpes simplex virus
